# Changes in perceived peripersonal space following the rubber hand illusion

**DOI:** 10.1038/s41598-023-34620-y

**Published:** 2023-05-12

**Authors:** M. Smit, H. C. Dijkerman, V. Kurstjens, A. M. de Haan, I. J. M. van der Ham, M. J. van der Smagt

**Affiliations:** 1 Department of Experimental Psychology, Helmholtz Institute, Utrecht University, Utrecht, The Netherlands; 2 Innovative Testing in Life Sciences and Chemistry, University of Applied Sciences Utrecht, Utrecht, The Netherlands; 3 Department of Health, Medical, and Neuropsychology, Leiden University, Leiden, The Netherlands

**Keywords:** Human behaviour, Cortex

## Abstract

Peripersonal space (PPS), the region immediately surrounding the body is essential for bodily protection and goal directed action. Previous studies have suggested that the PPS is anchored to one’s own body and in the current study we investigated whether the PPS could be modulated by changes in perceived body ownership. While theoretically important, this anchoring can also have implications for patients with altered body perception. The rubber hand illusion (RHI) is a way to manipulate body ownership. We hypothesized that after induction of a left hand RHI, the perceived space around the body shifts to the right. Sixty-five participants performed a landmark task *before* and *after* a left hand RHI. In the landmark task, participants had to determine whether a vertical landmark line was left or right from the center of a horizontal screen. One group of the participants was exposed to synchronous stroking, the other group experienced asynchronous stroking. Results showed a shift in space to the right (e.g. away from the own arm), but only for the ‘synchronous stroking’ group. These results suggest that the relevant action space becomes linked to the fake hand. Critically, subjective ownership experience did not correlate with this shift, but proprioceptive drift did. This suggests that multisensory integration of bodily information drives this shift in space around the body and not feelings of ownership.

## Introduction

Multiple senses give us feedback that our body belongs to us^1^. We can continuously see our body, we can feel touch through mechanoreceptors on our body, and we get feedback about the joint angle, muscle tension and muscle length regarding the location of our limbs. The integration of these senses, i.e. vision, touch and proprioception, contributes to creating awareness of our body and gives us the feeling that our body belongs to us, which is commonly referred to as body ownership^2^. Body ownership (BO), or more specifically arm ownership, can be experimentally manipulated; a well-known way to do so is the Rubber Hand Illusion (RHI)^3^. In the classic RHI a rubber hand is placed next to a subject’s own hand. Both hands (i.e., real and rubber) are stroked in synchrony at the same location, with only the rubber hand being visible. Watching the rubber hand being stroked, while simultaneously feeling the strokes on one’s own hand, causes the rubber hand to be attributed to one’s own body^4^. In order to integrate the new hand into the body representation, it is important that both the rubber hand and real hand are anatomically aligned^4–6^. In other words, they should be positioned in the same orientation and in parallel^3^—to each other or above one another^7^. Embodying the rubber hand as your own changes the sense of location of your own hand, that is, the perceived location of one’s own hand typically ‘drifts’ towards the rubber hand after inducing the illusion. This phenomenon is known as ‘proprioceptive drift’^3^. The RHI illustrates the plasticity of our body representation; it transiently changes how and where we perceive our hand. Manipulation of body ownership through illusions is not limited to the hand, but can also affect the foot^8^ and importantly the entire body^9,10^. However, in the current study we chose to use the rubber hand illusion as its potential effects on spatial perception are more easily assessed and have been reported before.

Several studies suggest that perception of space and body ownership are linked. Ocklenburg et al.^11^ investigated the influence of body ownership on pseudoneglect (i.e., a slight asymmetry of spatial attention *to the left* in healthy individuals) using a line bisection task^12^. Results indicated a reduction of pseudoneglect, but only after left hand RHI in high responders (i.e., participants who had a vivid rubber hand illusion). Interestingly, this reduction was not found in low responders (i.e., participants who showed a less strong illusion). These findings concur with a case displaying visuospatial neglect^13^. After the induction of a right hand RHI this patient showed a transient improvement of neglect on a cancellation task and line bisection; presumably the subjective midline shifted to the left allowing more space on the left side to be explored. Ocklenburg et al.^11^ proposed that the subjective midline shifts to the right after the left hand RHI (as the left rubber hand is closer to the body midline than the real left hand), and therefore stimuli in space also shift to the right (i.e., the score on a line bisection task). However, in their study they did not directly test the perceived direction of the body’s sagittal axis, i.e., the subjective midline. Moreover, the line bisection task involves a motor response (i.e. actively bisecting the line). Another task that is often used to determine space perception asymmetries is the landmark task^14^. In this task, a horizontal line is pre-bisected by a short vertical line, the landmark. The participant is asked to indicate whether the landmark is closer to the left or the right end of the horizontal line. Neglect patients generally indicate the left end of the line to be closer when the landmark is equidistant from both ends, suggesting that a lack of attention results in an underestimation of the perceived left half of the line^14,15^. Similarly, healthy participants show a slight overestimation of the extent of the left part of the line, consistent with pseudoneglect, on the landmark task^16,17^. The landmark task, in principle, is a visuospatial perceptual task, which can be performed without reference to the body. However, experimental studies have shown that the bias in landmark performance, depends on the distance of the lines from the body. When located in extrapersonal space, the leftward landmark bias is reduced or even absent^17–19^. Importantly, individuals with a hand amputation also show a distortion on the landmark task, with an underestimation of the line length on the amputation side^20^. This distortion was present only when the landmark task was performed in near space and not in far space. This suggests that performance on the landmark task near the participant involves mechanisms that differ from those used further away^21^. One important difference may be that near the body, the judgement of the location of the landmark also involves using a bodily reference frame.

In the current study we therefore examine whether altering the body representation using the rubber hand illusion influences performance on a purely perceptual spatial task, the landmark task. Specifically, our aim is to investigate whether a change in hand ownership caused by a left hand rubber hand illusion influences the landmark bias. The landmark task, which requires the participant to indicate whether a transection mark was located to the left or right of the center of a horizontal monitor was used *before* and *after* the RHI*.* As Ocklenburg et al.^11^ suggested that a shift in the subjective midline towards the right, as a consequence of feeling ownership over a left rubber hand, influenced the line bisection, we additionally measured the subjective midline by a subjective straight ahead pointing task *before* and *after* the RHI. This task required participants to point straight ahead, while blindfolded, to where they thought their bodily midline was. A between subject design was used with one group receiving synchronous stroking in the RHI set-up (presumed to induce the RHI), and a second group receiving asynchronous stroking (presumed to *not* induce the RHI). We anticipated that, because the rubber hand is located closer to the body midline (i.e. more to the right), inducing a left RHI in the synchronous group, would result in a rightward shift in body midline and hence a rightward shift in spatial perception as measured with the landmark task. We did not expect such a shift in the group which received asynchronous stroking.

## Methods

### Participants

In total, 65 undergraduate and graduate students participated in this study. The Embodiment Questionnaire^22^ was used to define to what extent participants experienced the illusion in both the synchronous stroking group (SG) and the asynchronous stroking group (AG). The synchronous group only received synchronous visuo-tactile stimulation when exposed to the rubber hand. In contrast, the asynchronous group only received asynchronous visuo-tactile stimulation (see Table 1 for demographic details of the two groups).

**Table 1:** Participant demographics for both the Synchronous Group, and Asynchronous Group (see text for details).

	N	Age	Gender F/M
Synchronous group	29	21.83 (2.00)	26/3
Asynchronous group	36	23.44 (5.15)	28/8

All participants were right-handed by self-report. Participants received course credits or 6 euros as a compensation for their time. They were naïve to the purpose of the study and a written informed consent was obtained from all individual participants prior to the experiment. This study was conducted in accordance with the standards of the declaration of Helsinki and was approved by the FETC of the Faculty of Social and Behavioural Sciences at Utrecht University.

### Design

**Figure 1:**
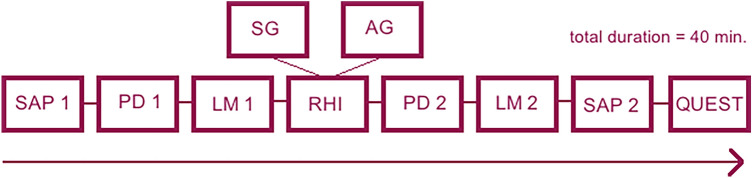
Timeline of the design: From left to right: SAP = straight ahead pointing, PD = proprioceptive drift, LM = Landmark, RHI = Rubber Hand Illusion, SG = synchronous stroking group, AG = asynchronous stroking group, 1 indicates the pre-measure, 2 indicated the post-measure. See Task/Stimuli for details of the tasks.

We conducted a pre-post between subjects design with type of stroking (i.e. either synchronously or asynchronously) as the between subjects factor. All participants completed several pre- and post-illusion measures (see task/stimuli below for detailed information for all the measures) in the exact same order (Fig. 1). Our primary outcome measure was an estimate of the point of subjective equality (PSE, see Analyses for details) generated from the pre- and post-Landmark tests. The test procedure started with the pre-measure of straight ahead pointing (SAP) followed by the pre-measure of the proprioceptive drift and the pre-session of the landmark-task (LM). Thereafter the RHI was induced (i.e. either synchronously or asynchronously depending on the group); the post-session of the proprioceptive drift, the landmark and straight ahead pointing followed the illusion respectively. In order to check whether the illusion was well executed, the proprioceptive drift (e.g. behavioral measure) measure followed the RHI immediately instead of the post-test of the Landmark. Ultimately, the embodiment questionnaire was administered.

### Task/stimuli/procedure

**Figure 2:**
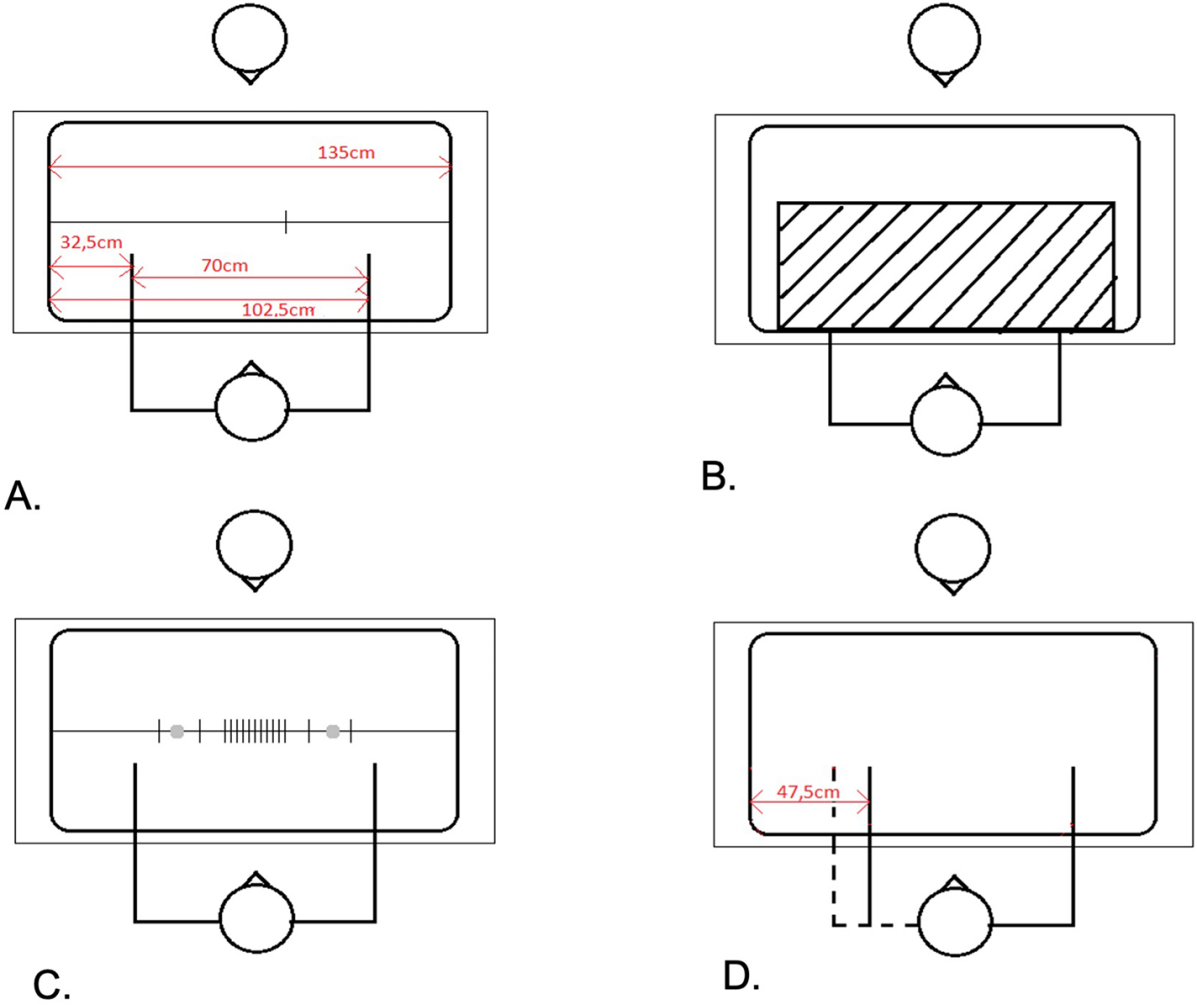
(**A**) Experimental set-up (not drawn to scale) and dimensions for the landmark task, top = experimenter, bottom = participant, one trial of the landmark is shown. (**B**) Set-up of proprioceptive drift with an occluder covering the lower arms (**C**) All possible landmarks (not drawn to scale). Only one of these landmarks was shown each trial. Each trial started with a static dot (either left or right from the center of the screen) that disappeared when the landmark appeared. (**D**). Hand positioning during the rubber hand illusion. Note that the dotted (real) arm was occluded by a black occluder. Only the added left rubber hand and the real right hand were visible to the participant.

Prior to the experiment participants were asked to remove all jewelry (i.e. rings and watches). The participant then seated oneself at the long end of a table, in front of a large horizontal screen (55inch) (see Fig. 2). The participant’s head was stabilized with a chinrest. For an overview of the set-up, see Fig. 2.

### Straight ahead pointing

The first experimental task was the straight ahead pointing (hereafter SAP, reflecting the subjective body midline), the participants placed their lower arms on the screen, in front of their body (wrists at 32.5 and 102.5 cm from the left side of the tablet, the wrists were equidistant from the body midline which was located at 67.5 cm from the left side of the tablet). The participants were instructed to point, with eyes closed to prevent feedback, with either their left or right hand at their own body midline and then in front of them where this midline would be on the monitor. This procedure was then repeated for the other hand. These locations were measured in cm from the left side of the tablet with a tape measure. For analyses, the SAP’s (in cm) were only used from the right hand as the left hand could have been influenced by the RHI, and the pre- and post-measures (cm) were included in the analysis. The duration of this task was about 3–5 min.

### Proprioceptive drift

After the SAP the *pre-measure* of the proprioceptive drift was conducted. A black occluder was placed over both hands to make them invisible, see Fig. 2b. The experimenter moved a stick from left to right (or right to left, random sequence) alongside the long end of the table. Participants were instructed to say stop when the stick was at the *felt* location of left or right index fingertip (again random sequence). The experimenter documented the exact location (by means of a tape measure) of the reported felt position. De left finger was always located 32.5 cm from the left edge of the box. The right index was 102.5 cm from the left edge of the box, see Fig. 2a. Thereafter, the occluder was removed. The pre- and post-measure (cm) were included in the analysis. The duration of this task was about 3–5 min.

### Landmark task

After the administration of the pre-test for proprioception, the pre-landmark task started. Participants were instructed to determine whether a landmark (i.e. vertical transection mark) was either left or right from the center of a grey line (Fig. 2c). In order to prevent feedback from previous landmark positions, each trial started with a static dot (500 ms) (Fig. 2c) appearing either on the left and right (alternately) from the center of the screen. Participants were instructed to look at these dots. Then a horizontal (dark grey) line across the whole width of the monitor (light grey background) was presented (Fig. 2a), followed by a 750 ms presentation of a vertical line (126 mm (200 pixels)) (i.e. the landmark; at a different location across the horizontal line in each trial; Fig. 2c). Locations of the vertical line were at (− 25.2, − 6.3, − 3.1, − 2.5, − 1.8, − 1.2, − 0.6, 0 (center), 0.6, 1.2, 1.8, 2.5, 3.1, 6.3, 25.2 mm; − 40, − 10, − 5, − 4, − 3, − 2, − 1 0, 1, 2, 3, 4, 5, 10, 40 pixels). To avoid aftereffects, a mask consisting of vertical lines was shown immediately after landmark presentation until the end of the trial. The participant indicated verbally whether the vertical line was located either to the *left* or *right* from the center of the screen. The experimenter pressed ‘A’ if the answer was ‘left’ and ‘L’ if the answer was right. Then the next trial started, for a total of 60 trials. Each of the 15 locations was presented 4 times in random order. Each participant’s data were fitted with a cumulative normal distribution function to generate estimates of the point of subjective equality (PSE) in Matlab; the location of the landmark where the participant was equally likely to determine it ‘left’ or ‘right’. The PSE, our primary outcome measure, was included in the analyses. The shift in PSE (before versus after the RHI) reflected the shift in space, hence peripersonal space. The duration of this task was about 10–15 min.

### Rubber hand illusion

After the first landmark session, the left arm was covered up by the occluder and the RHI was set up, see Fig. 2d. While the participant had his eyes closed, the rubber hand was placed in an anatomical congruent position 15 cm to the right of the real left hand^23^ and therefore 20 cm to the left of the body midline. To optimize the illusion, a cloth was placed over the shoulder of the participant. In the experimental condition, the illusion was established by stroking the index finger of the real and rubber hand simultaneously with a soft brush for 90 s, while the participant was visually focusing on the rubber hand. In the other group, the asynchronous group, the stroking was asynchronous: first the rubber hand was touched and then the real hand. Location and velocity of stroking were held constant.

After inducing the illusion, the rubber hand was removed and both real hands were covered by the occluder (Fig. 2b). The proprioceptive drift was now measured for the second time. This procedure was identical to pre-session. The occluder was then removed, so both hands were visible again, as in starting position (Fig. 2a). Then the landmark task started for the second time with the exact same procedure as in the pre-illusion session. Thereafter, the straight-ahead pointing task was performed and again the procedure was identical to the pre-illusion session.

### Embodiment questionnaire

To conclude the experiment, the participant filled out the Embodiment Questionnaire^22^. This questionnaire contained 10 items to measure the experience of the rubber hand illusion. The first three items measured the illusion experience, for example: ‘*It seemed like the rubber hand was my own’*, while the remaining seven items were control questions e.g. ‘*It seemed like I had more than two hands’.* The participant responded on a 11-point Likert scale with 0 = strongly disagree and 10 = strongly agree. The overall duration of the experiment was about 40 min.

### Analysis

For all our outcome-measures we used a Mixed ANOVA with *time* (pre-test versus post-test) as the within subject factor and synchrony (synchronous stroking versus asynchronous stroking) as between subjects factor. All p-values from post-hoc analyses were Bonferroni-corrected.

In addition, we also analyzed our data with a Bayesian mixed ANOVA, which uses a linear mixed model. We used Cauchy (uninformative) priors on effect size^24,25^. Thus, next to the frequentist approach we report Bayes factors which yields the probability of a model given the data (i.e., a certain combination of effects) relative to a null model (i.e., no effects), that is, values larger than 1 are in favor of H1. Bayes Factors (BF) that provide evidence in favor of the null model are abbreviated as BF_01_, Bayes Factors that provide evidence in favor of a difference are abbreviated as BF_10_. Since the Bayesian approach can quantify evidence for both directions (e.g. evidence for H1 *and* evidence for H0), it allows evaluating null effects, which is not the case in the classical frequentist approach^24^.

## Results

### Subjective ownership in the Synchronous stroking Group (SG) and Asynchronous stroking Group (AG)

**Figure 3:**
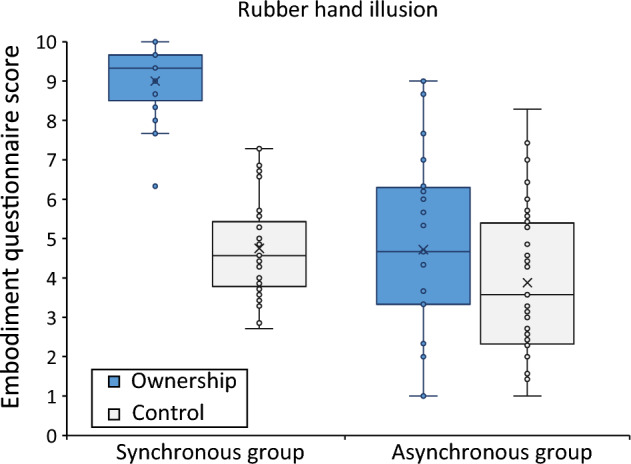
Boxplot of the data on the ownership questionnaire (see text for details). The panel shows the average score on the ownership scale (question 1–3) and control scale (question 4–10) for the Synchronous stroking Group and Asynchronous stroking Group (including individual scores). Error bars represent the 95% confidence intervals. (see also Supplementary Table 1).

In total we tested 65 participants (36 in the asynchronous stroking group and 29 in the synchronous stroking group). A Shapiro Wilk test showed that data approximated a normal distribution, except the ownership scale (*p* < 0.001), all other *p-values* ≥ 0.154. A mixed ANOVA revealed a main effect of *subscale*, *F*(1,63) = 136.07, *p* < 0.001, partial η^2^ = 0.684, indicating a higher score for the ownership-subscale than the control-subscale (see Fig. 3 and Supplementary Table 1). There was also a between *groups*-effect, *F*(1,63) = 47.47, *p* < 0.001, partial η^2^ = 0.430, indicating a higher score for the SG than for the AG. Additionally, we found an interaction between *subscale* and *group F*(1,63) = 58.28, *p* < 0.001, partial η^2^ = 0.481. Similarly, Bayesian analyses revealed that the highest posterior model probability (P(M) = 0.2, P(M|data) = 3.586e + 23) was for the model that included main effects for *subscale* and *group* and the interaction effect *subscale* x *group*, this was considered an extreme effect*. Post-hoc* Bonferroni corrected t-testing revealed that this interaction effect was driven by the difference between the ownership-scale of the SG and AG, *t*(63) = − 9.438, *p* < 0.001 Cohen’s *d* = − 2.355 (BF_10_ = 4.29e + 10, which is classified as extreme evidence in favor of a difference) and not the control subscale, *t*(63) = − 2.116, *p* = 0.076 Cohen’s *d* = − 0.528 (however BF_10_ = 1.64, which may be classified as slight anecdotal evidence *for* a difference).

### Proprioceptive drift (PD) for the SG and AG

**Figure 4:**
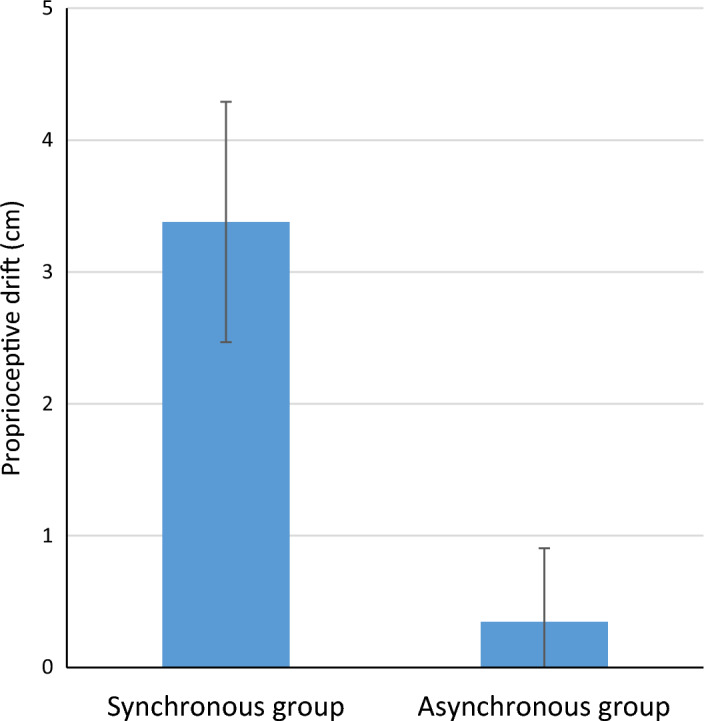
Average estimates in cm of proprioceptive localisation (i.e. difference between pre- and post-illusion) for the synchronous stroking and asynchronous stroking groups for the left index finger. Error bars represent standard error of the mean (see also Supplementary Table 1).

For the proprioceptive drift a Shapiro Wilk showed that all data, except for the pre-measure (*p* = 0.041) of the SG, approximated a normal distribution, all other *p-values* ≥ 0.235. The mixed ANOVA revealed a main effect of time *F*(1,63) = 13.161, *p* = 0.001, partial η^2^ = 0.173, and an interaction of *time* (pre vs. post) x *group* (SG vs. AG) *F*(1,63) = 8.713, *p* = 0.013, partial η^2^ = 0.121 (see also Fig. 4 and Supplementary Table 1). Bayesian analyses revealed that the highest posterior model probability (P(M) = 0.2, P(M|data) = 0.884) was for the model that included main effects for *time* and *group* and the interaction effect *time* x *group.* The Bayes Factor* (*BF_10_) was 30,588.791, which is considered as extreme evidence in favor of this model, indicating that the type of stroking (i.e., synchronously or asynchronously) had a differential impact on proprioceptive drift (inclusion Bayes factor for the interaction: 3715.825). To further test this, we applied both a Paired Samples T-test and a Bayesian Paired Samples T-test to compare the pre- and post-sessions for each group. These analyses revealed a significant difference between the pre- and post-session *t*(28) = − 3.707 *p* < 0.001, Cohen’s *d* = − 0.688, with a Bayes Factor of 36.099 for the SG, which is considered as very strong evidence for a difference (Suppl Fig. 1). As expected for the AG, the pre- and post-session did not differ, *t*(35) = − 0.0622 *p* = 0.538, Cohen’s *d* = − 0.104. The Bayes Factor was 0.214 indicating moderate evidence *against* a difference between the pre- and post-test. Results revealed that participants indeed drifted proprioceptively towards the rubber hand after synchronous stroking, indicating the RHI was well induced.

### Landmark task in the SG and AG

The data of each individual participant was fitted using a cumulative normal distribution function to generate estimates of the point of subjective equality. Overall, the R^2^ showed a reasonable to good fit (SG mean R^2^ = 0.69 pre-illusion; mean R^2^ = 0.70 post-illusion; AG: mean R^2^ = 0.68 pre-illusion; mean R^2^ = 0.67 post-illusion). The landmark estimates (i.e. PSE) were analyzed using both a mixed ANOVA and a Bayesian Mixed ANOVA with *time* (pre versus post) x group (synchronous versus asynchronous) mixed ANOVA.

**Figure 5:**
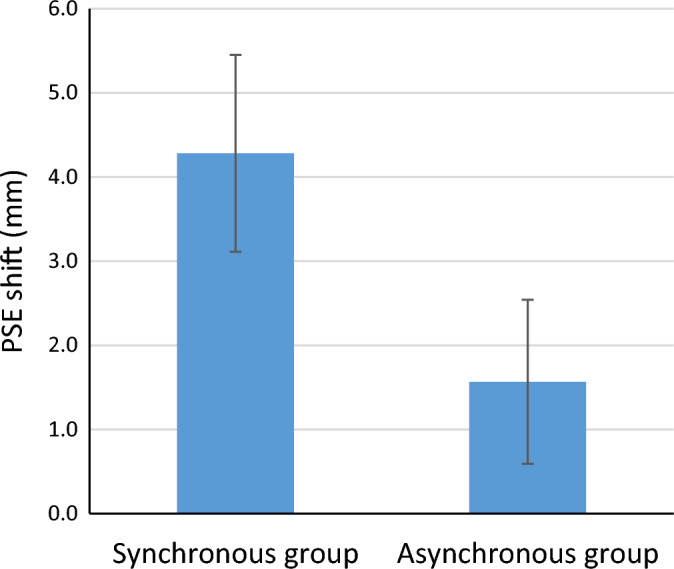
Average shifts in point of subjective equality (PSE) on the landmark task (i.e. difference between pre- and post-illusion) for the synchronous stroking and asynchronous stroking groups. The PSE is depicted in mm for convenience but has been analyzed in pixels. The error bars depict the standard error of the mean (see also Supplementary Table 1).

A Shapiro Wilk test showed that the data was normally distributed, all *p* ≥ 0.235. A mixed ANOVA revealed a main effect of *time F*(1,63) = 14.951, *p* < 0.001, partial η^2^ = 0.184, and a near significant interaction between *time* and *group F*(1,63) = 3.219, *p* = 0.078, partial η^2^ = 0.049 (see also Fig. 5 and Supplementary Table 1). Bayesian analyses revealed the highest posterior model probability (P(M) = 0.2, P(M|data) = 0.423) was for the model that only included the main effect of *time* (BF_10_ = 45.583)*.* The BF_10_ for the model that included all the effects (main and interaction) was 15.664, which is still considered as strong evidence in favor of this model, however it is not the best model given the data. We however decided to apply subsequent t-tests to compare the average PSE of the participants before the RHI to the average PSE after the RHI in the SG, and the AG. This difference between the pre- and post-session was only statistically significant for the SG, *t* (28) = − 3.653 *p* = 0.001, Cohen’s *d* = 0.678 (BF_10_ = 31.879, which is classified as very strong evidence in favor of a difference between pre- and post), but not for the AG, *t*(35) = − 1.606, *p* = 0.117 Cohen’s *d* = 0.268 (BF_10_ = 0.576, which is classified as anecdotal evidence against a difference between pre-and post; see Suppl Fig. 2).

### Straight ahead pointing for the SG and AG

**Figure 6:**
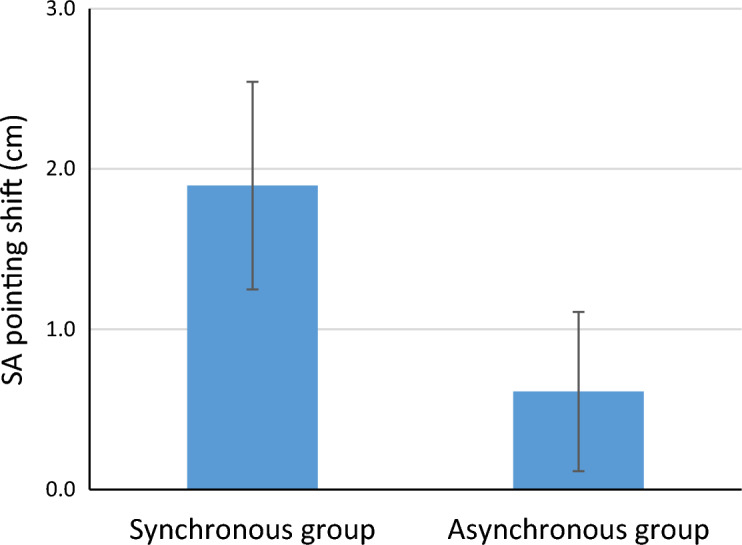
Average shifts in pointing straight ahead (i.e. difference between pre- and post-illusion) for the synchronous stroking and asynchronous stroking groups. The error bars depict the standard error of the mean (see also Supplementary Table 1).

As the position of the left hand was in influenced by the RHI, we only analysed the data for SAP for the right hand. A Shapiro Wilk test showed that data was not normally distributed for both the pre- and post-session of the AG (*p* = 0.009, and *p* = . 047, respectively), and for the post condition of the SG (*p* = 0.005). A Wilcoxon signed-rank test showed a significant difference between pre- and post-illusion SAP for the SG group *Z* = − 2.76, *p* = 0.006 (see also Fig. 6 and Supplementary Table 1). This difference was not significant for the AG group, *Z* = − 1.53, *p* = 0.13. Bayesian analyses (see suppl Fig. 3) revealed moderate evidence in favor of a pre- post difference for the SG (BF_10_ = 6.39) and anecdotal evidence in favor of the H0 hypothesis for the AG (BF_01_ = 2.786).

All in all, we find a significant shift in peripersonal space (i.e. shift in PSE) to the right only after synchronous stroking. This shift to the right is also apparent in the proprioceptive drift and in straight ahead pointing task. Our next step was to test whether our primary outcome measure, the PSE, which reflected perception of the peripersonal space, correlated with any of the secondary outcome measures. In the next paragraph, we tested whether the shift in space was driven by feelings of ownership (ownership-scale of the questionnaire), proprioceptive drift, and lastly if the shift in space also correlated with a shift in subjective body midline.

### Correlations between the shift in PSE, subjective ownership, shift in proprioceptive drift, and shift in straight ahead pointing.

All participants (SG and AG together) were used to test whether there was a relation between the shift in the PSE (pre-post) and subjective ownership, proprioceptive drift (post–pre) for the left hand, and straight-ahead pointing (pre-post) with the right hand. The ownership scale, the proprioceptive drift (post–pre) and straight-ahead pointing (pre-post) deviated from normality (*p* < 0.001; *p* = 0.014 and *p* = 0.006 respectively), the shift in PSE was normally distributed *p* ≥ 0.316. Kendall’s tau statistic was used for all the correlational analyses. We found a significant correlation between the shift in PSE and the shift in the proprioceptive drift, *τ*_b_ = 0.239, *p* = 0.007, indicating that the larger the proprioceptive drift towards the rubber hand (i.e. shift towards the right), the more the participants shifted to the right in space. The analyses also revealed a significant correlation between the shift in PSE and the shift in body midline as measured with the right-hand SAP *τ*_b_ = 0.235, *p* = 0.007, indicating that the shift in space to the right was related to a rightward shift in the subjective midline on the body. The analyses did not reveal a significant correlation between the shift in PSE and subjective feelings of ownership *τ*_b_ = 0.10, *p* = 0.26. Intriguingly, subjective ownership did not correlate to the shift in PSE, but was significantly correlated to the proprioceptive drift, *τ*_b_ = 0.242, *p* = 0.007. Furthermore, while proprioceptive drift and SAP were both significantly correlated with a shift in PSE, they did not correlate with each other, *τ*_b_ = 0.109, *p* = 0.224.

## Discussion

In the current study we aimed to test the flexibility of our peripersonal space with the rubber hand illusion. Specifically, we tested whether performance on the landmark task can be modulated by a change in ownership caused by the rubber hand illusion. To investigate this question, two groups of participants performed a landmark test *before* and *after* the RHI (e.g. 90 s of multisensory stimulation). The landmark task required the participant to indicate whether a transection mark was located to the left or right of the center of a screen. We divided our participants in two groups; one group experienced synchronous stroking on the left hand, and the other group experienced asynchronous stroking on the left hand. We expected that the bias on the landmark task to be shifted to the right as a consequence of feeling ownership over a left rubber hand. We indeed found a shift to the right in PSE following illusion induction, and only for the ‘synchronous stroking’ group. These findings concur with previous findings of Ocklenburg et al.^11^. In a somewhat different experiment, Ocklenburg et al.^11^ found that “high responders” (e.g. individuals who experienced the RHI vividly) as opposed to low responders (e.g. individuals who reported a low illusion score) shifted (i.e. the perceived space) to the right on a line bisection task after left-sided RHI. The line bisection task is slightly different from the landmark task, as it requires an active motor response, but the rationales of the tasks are very similar; both represent a shift in space. The authors suggest that for the high responders the rubber hand was integrated in their body image, which was not the case for low-responders. In comparison to Ocklenburg et al.^11^ the shift that we observed was similar, albeit slightly smaller in magnitude. We believe that the magnitude of our shift was attenuated by the duration of inducing the illusion, which was only 90 s, relatively short compared to Ocklenburg and colleagues’^11^ 180 s. Also, in their study, the Line Bisection task followed the inducement of the illusion immediately, and in our design, we first measured the proprioceptive drift for both hands and thereafter we performed the landmark for ten minutes. In hindsight, we believe that the set-up of our post-illusion landmark-task also attenuated the effects: We now kept the pre- and post-landmark-measures identical, so if we would find an effect (i.e. shift in PSE) it could only be attributed to the type of stroking. Although this is probably the case, viewing your own right hand, however, in the post-landmark task might have provided visual feedback of one’s body midline, and consequently decreased the magnitude of the rightward shift in space. Finally, another difference between our study and that of Ocklenburg^11^ is that we used synchronous and asynchronous stroking in two different groups to induce differences in ownership over the rubber hand, while Ocklenburg depended on individual difference in sensitivity to the RHI to make two groups. As a consequence, it is likely that our synchronous group contains individuals who did not experience ownership over the rubber hand and the asynchronous group individuals who did experience ownership of the rubber hand (see Fig. 3). This may have resulted in smaller effects on the landmark task. Nevertheless, overall, there was a robust difference in RHI effects on the questionnaires and proprioceptive drift between the two groups.

It seems warranted that changes in the representation of the body (e.g. embodying a rubber hand) can, at least transiently, change how the space surrounding the body (the peripersonal space) is perceived. Literature suggests a close and dynamic relationship between the two representations at a neural and behavioral level. These accounts were first demonstrated at a neural level in monkeys; bimodal neurons in the multisensory brain areas (e.g. premotor and parietal areas) respond to both tactile stimuli *on* the monkey’s limb and visual stimuli *nearby* the limb^26–28^. Numerous behavioral studies in humans using different kinds of bodily illusions have found that spatial characteristics of peripersonal space can be modulated and that boundaries can be extended to include a fake or virtual arm^6,29–32^. The general idea is that embodying a fake arm after multisensory stimulation can alter the spatial features of the receptive fields of multisensory neurons in such a way that now the fake or virtual body part is included in the body image (see Blanke et al.^33^ for an insightful discussion on this topic). Although we are not measuring the boundaries of peripersonal space per se, our set up differs slightly from the studies just mentioned, we do believe that if an arm is integrated in the body representation, it can shift the perceived body space and objects presented in that body space.

Intriguingly, the rightwarded shift was not related to the subjective feeling of ownership, thus more explicit accounts (e.g. via a questionnaire) of experienced body ownership per se do not drive these changes in space. However, the shift in space was related to proprioceptive drift, which is an implicit measure of the shift from the real to the artificial hand and to a shift in pointing straight ahead with the non-illusion right hand. During the proprioceptive drift, the visual input becomes more dominant than the proprioceptive input. In order for our brain to reconcile this, the visual dominance shifts the perceived localization towards the *seen* rubber hand^6^, and thus distorts our position sense. The term ‘dominant’ might be misleading here, since especially adults (as opposed to young children) integrate *all* the incoming senses in an optimal way or “statistical optimal fashion”, i.e. weigh the reliability of visual, proprioceptive and tactile signals in a given task^34^. Moreover, adults seem to give more weight to visual input in the horizontal direction (i.e. left/right), while more weight is given to proprioception in depth perception (i.e. near/far)^35,36^, commonly referred to as the direction dependent weighing account^35^. The finding that this shift in proprioceptive drift in our study correlates to our shift in space (e.g. PSE) is then not surprising: both underlying mechanisms of these outcome measures are visuo-spatial in nature (and showed a shift from left to right).

In contrast, the questionnaire is a more indirect and cognitive measure. Thus, we found a correlation between the shift in PSE and the proprioceptive drift, but no correlation between the shift in PSE and the ownership questionnaire. One would conclude that the drift and the ownership questionnaire then measure different aspects of body ownership. However, we actually did find a correlation between the questionnaire and the proprioceptive drift, indicating overlap between the underlying mechanisms. Recent accounts^37^ using the mirror illusion also concluded that the “same integration or matching processes between visual and proprioceptive feedback could be used to evoke proprioceptive drift, feeling of ownership, and agency”, although see Rhode et al.^38^ for a discussion on the dissociation between subjective ownership and proprioceptive drift. Thus, some overlap is required between the underlying mechanisms of these measures, but they were not equally related to the shift we found in peripersonal space. To what extent and in what way they do overlap remains inconclusive^37^.

Another contributing factor to the shift in PSE on the landmark task, appears to be the perception of the subjective body midline. In his study on the effect of the RHI on pseudo-neglect, Ocklenburg et al.^11^ suggested that the shift in line bisections might be related to a shift in subjective experience of this body midline. This idea was assessed in the current study using the straight-ahead pointing task. Indeed, we found that the subjective body midline had shifted more to the right in the SG compared to the AG. Moreover, overall, the shift in straight-ahead pointing following the illusion also correlated with the shift in space as measured using the landmark task. Both these findings are consistent with the idea that the RHI induces a shift in subjective perception of the body midline, which affects perception of space around the body. However, intriguingly, while both the proprioceptive drift in left hand location and the change pointing straight-ahead (using the non-illusion right hand) were correlated with the change in PSE on the landmark task, they did not correlate with each other. This suggests that the shift in subjective body midline is not necessarily linked to the change in perceived left-hand position following the RHI. Rather, the RHI might affect the spatial representation that is linked to the proprioceptive localization of the hands (i.e. hand-centered) and that of the torso (the subjective midline (e.g. straight-ahead judgments) independently and both contribute to a shift in perceived landmark location. Future studies should confirm this idea.

To conclude, the present study, combined with previous studies indicates that changes in bodily processing can modulate the perceived space around the body. This change seems to stem from a shift in proprioceptive localization, rather than subjective feelings of ownership and from a change in subjective body midline. The findings presented in this manuscript are of particular interest for certain groups where proprioceptive input is compromised, which frequently occurs after stroke^39^. Our results suggest that not only bodily information will be differentially processed (i.e. suboptimal multisensory integration) as was recently found^40,41^, but also the space around the body. Future studies should thus also focus whether the region around the body is impacted by disturbances in body ownership.

## Supplementary Information

Supplementary Information 1.

Supplementary Information 2.

## Data Availability

The datasets generated and analysed during the current study are available from the corresponding author on request.
