# Supplementary material for: Changes in perceived peripersonal space following the rubber hand illusion
Source: Sci Rep. 2023 May 12;13:7713. doi: 10.1038/s41598-023-34620-y (PMC10182095; doi:10.1038/s41598-023-34620-y)

SUPPLEMENTARY FIGURES.

Suppl fig. 1. Sequential plots for the synchronous stroking group (left panel) and the asynchronous stroking group (right panel) for proprioceptive drift. The current n provides very strong evidence in favor of the alternative hypothesis (H1; a difference between pre and post) in the synchronous group, whereas evidence was moderate against a difference (H0) between the pre- and post-session in the asynchronous stroking group.

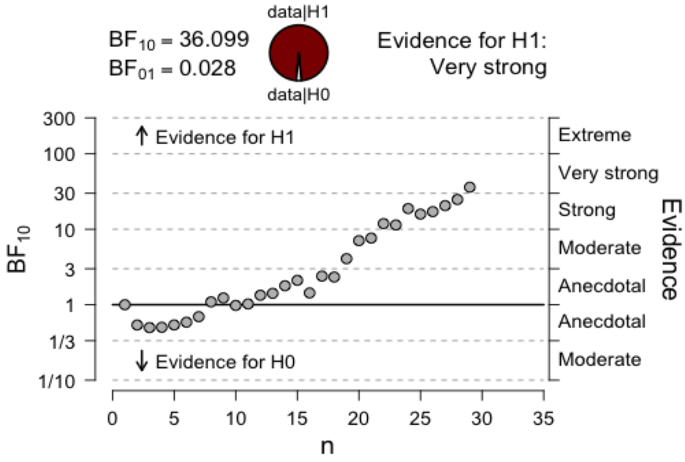

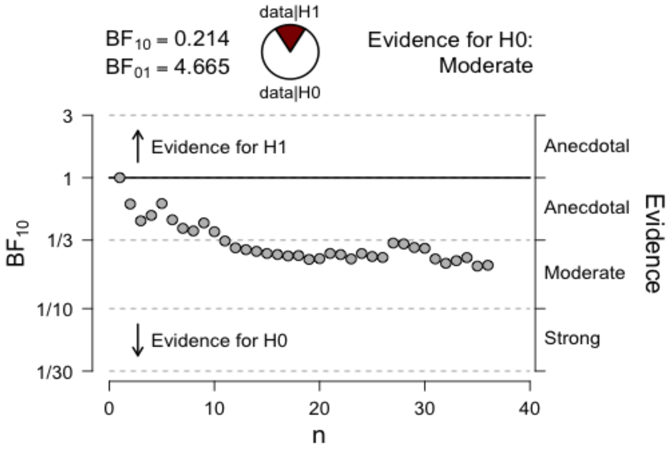

Suppl Fig. 2. Sequential plots for the synchronous stroking group (left panel) and the asynchronous stroking group (right panel) for the landmark task. The current n provides very strong evidence in favor of the alternative hypothesis (H1; implying a difference between pre- and post-session) in the synchronous group, whereas evidence is anecdotal in favor of no difference between the pre- and post-session in the asynchronous stroking.

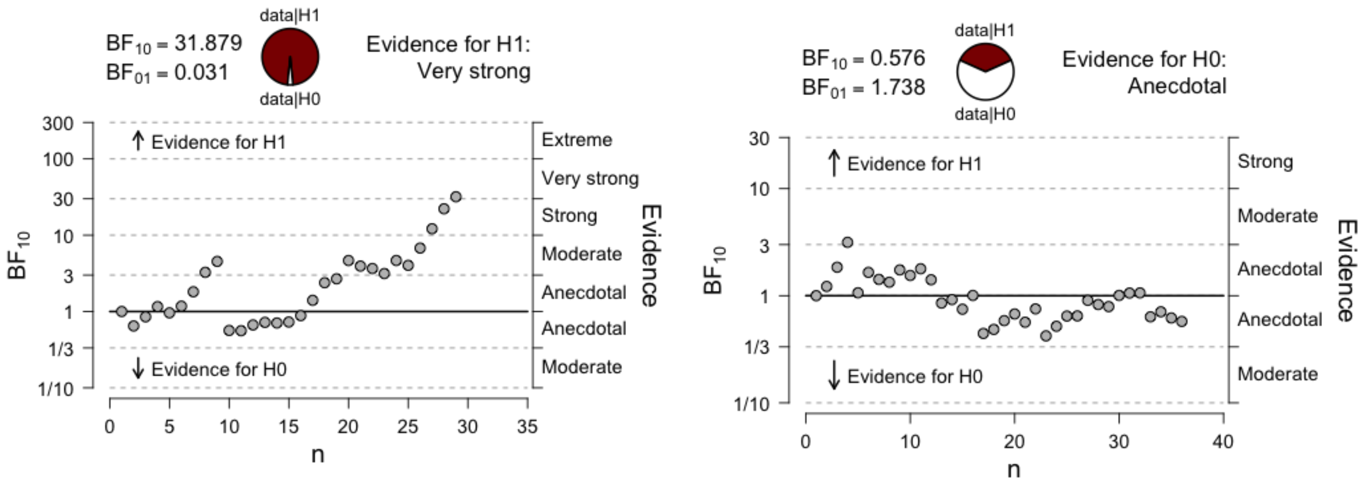

Suppl figure 3. Sequential plots for the SG (left panel) and the SG (right panel) for straight ahead pointing. The current n provides moderate evidence in favor of the alternative hypothesis (H1; implying a difference between pre- and post-session) in the synchronous group, whereas evidence is anecdotal in favor of no difference between the pre- and post-session in the asynchronous group.

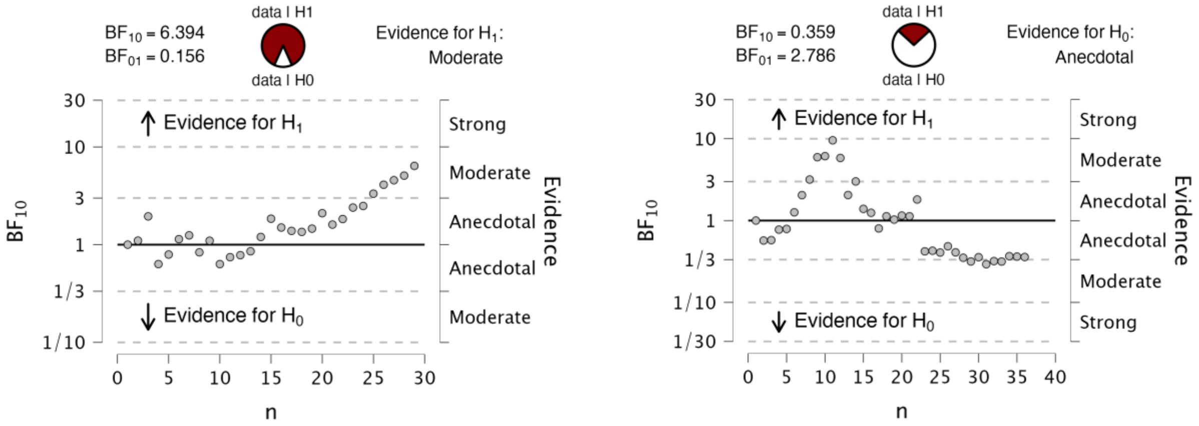

Supplement: Supplementary file 1 — Supplementary Information 1. [file 41598_2023_34620_MOESM1_ESM.pdf]
