# Supplementary material for: Changes in perceived peripersonal space following the rubber hand illusion
Source: Sci Rep. 2023 May 12;13:7713. doi: 10.1038/s41598-023-34620-y (PMC10182095; doi:10.1038/s41598-023-34620-y)
Supplement: Supplementary file 2 — Supplementary Information 2. [file 41598_2023_34620_MOESM2_ESM.pdf]

	Questionnaire		Proprioceptive drift		Landmark task		Straight ahead pointing	
	Ownership	Control	Before	After	Before	After	Before	After
Synchronous group	9.00 (0.86)	4.75 (1.27)	38.62 cm (3.91)	42.00cm (4.70)	-0.79 mm (7.88)	3.49 mm (8.61)	67.64 cm (2.80)	69.53 cm (3.60)
Asynchronous group	4.77 (2.28)	3.88 (1.91)	36.40 cm (3.90)	36.75 cm (3.87)	1.18 mm (7.28)	2.74 mm (7.11)	68.78 cm (2.66)	69.39 cm (3.06)

Supplementary table 1: Descriptives of all the dependent variables analysed. Proprioceptive drift was measured from the left edge of the box. The left index finger was located at 32.5cm. The landmark task was measured from the centre of the screen in mm. A negative value indicated a leftward bias. The straight ahead pointing was measured in cm from the left side of the tablet. The body midline was located at 67.5 cm.
